# A Comprehensive Review of Micro/Nano Precision Glass Molding Molds and Their Fabrication Methods

**DOI:** 10.3390/mi12070812

**Published:** 2021-07-12

**Authors:** Md. Ali Asgar, Jun Kim, Muhammad Refatul Haq, Taekyung Kim, Seok-min Kim

**Affiliations:** 1 Department of Computer Science and Engineering, Chung-Ang University, 84 Heukseok-ro, Dongjak-gu, Seoul 06974, Korea; asgar@cau.ac.kr; 2 Department of Mechanical Engineering, Chung-Ang University, 84 Heukseok-dong, Dongjak-gu, Seoul 06974, Korea; zuhn@cau.ac.kr (J.K.); refat@cau.ac.kr (M.R.H.); 3 Department of Systems Engineering, University of Texas at Dallas, Richardson, TX 75080, USA

**Keywords:** micro/nano-precision glass molding, mold materials, surface roughness, glass transition temperature, anti-adhesion coating

## Abstract

Micro/nano-precision glass molding (MNPGM) is an efficient approach for manufacturing micro/nanostructured glass components with intricate geometry and a high-quality optical finish. In MNPGM, the mold, which directly imprints the desired pattern on the glass substrate, is a key component. To date, a wide variety of mold inserts have been utilized in MNPGM. The aim of this article is to review the latest advances in molds for MNPGM and their fabrication methods. Surface finishing is specifically addressed because molded glass is usually intended for optical applications in which the surface roughness should be lower than the wavelength of incident light to avoid scattering loss. The use of molds for a wide range of molding temperatures is also discussed in detail. Finally, a series of tables summarizing the mold fabrication methods, mold patterns and their dimensions, anti-adhesion coatings, molding conditions, molding methods, surface roughness values, glass substrates and their glass transition temperatures, and associated applications are presented. This review is intended as a roadmap for those interested in the glass molding field.

## 1. Introduction

The fabrication of micro/nano devices on glass substrates has gained increasing attention due to their excellent chemical resistance, scratch resistance, mechanical stiffness, and superior optical characteristics (e.g., high transmittance, low ultraviolet (UV) absorption, and high refractive index). Numerous readily scalable methods have been developed to pattern micron- and submicron-sized features onto glass material, and these can generally be classified into (i) wet or dry etching methods using photolithographed or nanosphere lithographed barrier patterns [1,2,3,4], (ii) micromachining methods, which include laser machining, micro-drilling, and powder blasting [5,6,7,8,9], and (iii) micro/nano glass molding methods [10,11,12]. Of these processes, micro/nano-precision glass molding (MNPGM) has gained significant attention because of its simplicity, cost-effectiveness, flexibility, cleanliness, and most importantly, environmentally friendliness. Glass molding was first developed by Kodak in 1940 in the US [13]. Although glass molding and hot embossing are synonymous and are often used interchangeably, there exists an obvious difference. For example, hot embossing is a general term that includes micro/nano replication of polymers as well as glass materials from a mold material. In contrast, glass molding or micro/nano glass molding deals only with the duplication of micro/nanostructures only on the glass materials from the mold material. MNPGM is typically employed in general optics related to light manipulation, which is important for a wide range of commercial products, including camera lenses, CD and DVD optical lenses, fiber connectors, microfluidic systems, concentrator photovoltaic (CPV) systems, antireflection products, display systems, optical data storage devices, and image processing products.

**Figure 1:**
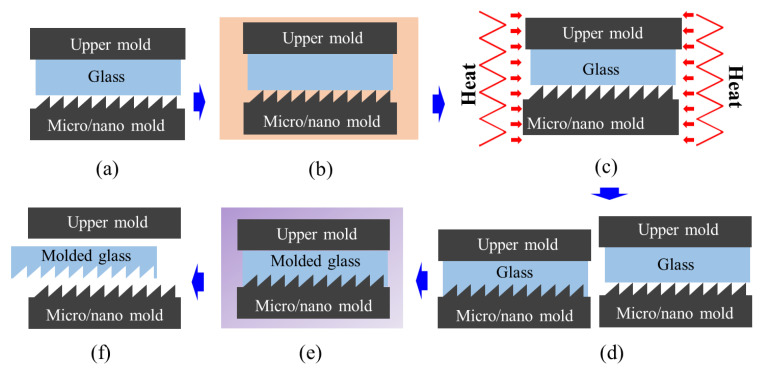
Schematic illustration of the glass molding process: (**a**) loading of the glass material between lower and upper molds, (**b**) purging of the chamber, (**c**) heating of the molding chamber to the molding temperature, (**d**) clamping and pressing of the glass material against the upper and lower molds, (**e**) cooling of the molded glass, and (**f**) unloading of molded glass.

The principal components of the glass molding process are the glass material, molding machine, and mold insert. The main components of the molding machine are the molding chamber, heating system, and pressing unit [14,15,16]. In principle, glass molding is a direct single-step process with three major phases: heating, pressing, and cooling. A typical glass molding process is illustrated in Figure 1. First, a glass perform is carefully positioned onto the lower mold, which is followed by the purging of air to avoid the oxidation of the glass and mold at high temperatures. Next, the mold and glass material are heated together to a predefined molding temperature by a heating source. Then, the glass is clamped between the lower and upper molds and then compressed to a specific molding pressure. Finally, the glass and the mold are cooled to room temperature before the glass is released from the molds. Typically, the cooling cycle is carefully controlled because cooling may lead to internal thermal stress, which can change a number of important properties, including the refractive index and Abbe number [17,18,19,20]. Based on the glass transition temperature (*T_g_*), above which the glass material becomes viscous fluid [21], optical glass materials can be categorized as (i) ultra-low *T_g_* glass (*T_g_* ˂ 400 °C), (ii) low *T_g_* glass (400 °C ˂ *T_g_* ˂ 620 °C), or (iii) high *T_g_* glass (*T_g_* ˃ 620 °C) [13].

In the glass-molding process, the molding temperature, pressure, pressing time, and cooling rate need to be optimized in advance. Thus, a number of theoretical and experimental studies have investigated the optimization of the molding parameters for glass molding [22,23]. In an ideal case, the quality of the molded glass optics depends solely on the quality of the mold under these optimal imprinting conditions. For this reason, mold selection, design, and fabrication have also been a major focus of research, and various optimized mold materials with desired pattern geometries have been reported. For instance, He et al. [24] reported the fabrication of a silicon (Si) mold with microstructures fabricated using lithography and reactive ion etching (RIE) for optical glass molding. In addition, Choi et al. [25] developed a tungsten carbide (WC) mold with microlens (ML) patterns via a sintering and polishing process, while Xie et al. [26] studied a nickel–phosphorous (Ni–P) alloy mold with a microgroove structure produced using electroforming and micromachining techniques. Lee et al. [27] described the fabrication of a glassy carbon (GC) mold with a microchannel pattern that was produced using micromachining in an electrolytic medium. Zhou et al. [28] reported a silicon carbide (SiC) mold with a microlens array (MLA) using micromachining and RIE. The fabrication techniques used to produce these molds have certain advantages and disadvantages in terms of optimizing the aspect ratio, surface roughness, geometric texture, and, most importantly, processing costs of the molds.

To date, several comprehensive reviews have been reported that summarize the key issues associated with the precision glass molding process [29], which are issues related to the replication of non-spherical glass lenses [30], theoretical and technological advancement of current glass molding [31], chalcogenide glass for infrared (IR) optics [32], and the manufacturing of optical micro-components via glass molding [33]. However, despite the mold being an indispensable component of micro/nano glass molding, there has been no review of the fabrication of these molds. Thus, this review article aims to summarize the latest advances in molds for MNPGM and their fabrication methods. After a brief discussion of the key requirements for MNPGM molds, common mold materials and their fabrication methods are reviewed individually. Various characteristics of these materials, such as the surface finish, molding temperature, and coating requirements, are addressed. In each section, a table summarizing the mold fabrication methods, mold patterns and their dimensions, anti-adhesion coatings, molding conditions, molding methods, surface roughness values, glass substrates and their glass transition temperatures, and their applications is presented.

## 2. Mold Materials for Precision Glass Molding

The selection of mold inserts has a pivotal impact on glass imprinting at the micro/nanoscale. In order to achieve the desired replication quality, the mold should be fabricated with high accuracy and efficiency. Molds for glass imprinting should also meet the following requirements:

- Higher thermal conductivity and lower thermal expansion compared to the glass used for molding experiments
- Workability under high pressure and ability to withstand repeated heating and cooling
- High stiffness and no adhesion to the glass substrate at high temperatures
- Low surface roughness so that it is suitable for optical applications.

**Figure 2:**
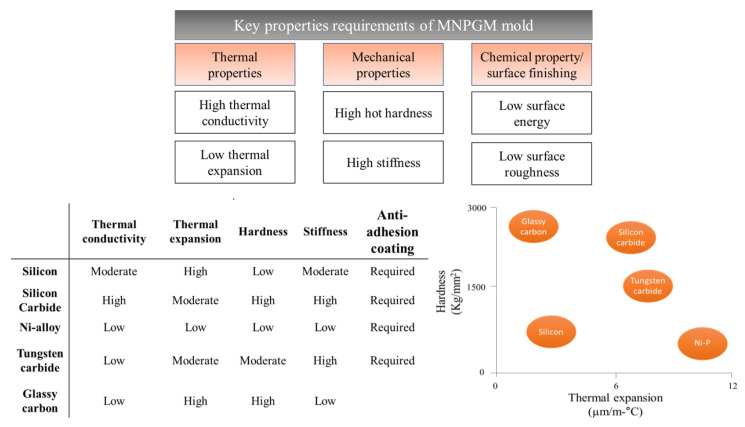
Important properties required of MNPGM molds and a comparison of their material properties.

Relationships among different properties of various molds are displayed in Figure 2. Currently, there is a wide variety of mold insert techniques available for micro/nano glass imprinting. A mold can be fabricated in many ways, and each fabrication technique has its advantages and disadvantages in terms of design flexibility, fabrication simplicity, and cost-effectiveness. The present section summarizes various molds and their fabrication approaches.

### 2.1. Si Molds

Si has been commonly utilized as a mold material for various types of micro/nano molding using polymers [34], aluminum (Al) [35], and glass [36]. Due to its material properties, processing on Si substrates is well established and fully accessible, making Si a particularly promising mold material. Versatile micro/nanostructures, including micrograting patterns, MLAs, kinoform lenses, and diffractive optical elements (DOEs), have been fabricated on Si mold surfaces for imprinting onto glass substrates. To fabricate micron or submicron patterns, subtractive approaches such as dry or wet etching, direct machining, or a combination of both etching and direct machining are generally utilized. For example, Saotome et al. [36] studied a V-groove structure with widths of 100 nm to 2.0 µm on a Si mold surface produced using electron beam lithography (EBL) and anisotropic etching. Then, these microgroove arrays were replicated from the Si mold on a glass specimen via hot embossing. Using lithography and etching, micro/nanostructures with vertical sidewalls can be fabricated.

**Figure 3:**
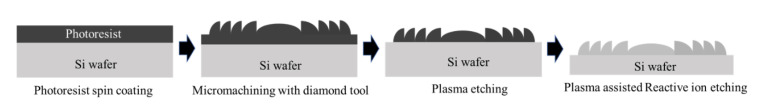
Schematic illustration of the micromachining process on a Si wafer.

Although widely used for the fabrication of various micro/nanostructures, conventional lithography and etching processes are limited to producing two-dimensional (2D) structures [37]. To produce accurate three-dimensional (3D) structures, intricate planar approaches such as bulk micromachining and surface micromachining are required [38,39,40]. For this reason, another subtractive technique, ultraprecision tuning, has been employed, in which the material is removed by keeping the cutting tool stationary and rotating the Si workpiece. Complex microstructures, such as MLAs and Fresnel lenses, have been successfully manufactured using precision machining. For instance, Li et al. [41] utilized ultraprecision single-point turning to develop an MLA with a diameter of 60 µm on a Si wafer for thermal imprinting on glass. However, micromachining on Si wafers is challenging because Si is a stiff and brittle material. To overcome this issue, micromachining on a SU-8 photoresist pattern followed by dry etching (shown in Figure 3) was employed by Chen et al. [42]. A Fresnel lens pattern was successfully transferred from the micromachined photoresist to the Si wafer through controlled plasma-assisted RIE. Then, this Fresnel lens was transcribed to the glass material using an isothermal glass molding process.

Examples of glass imprinting using Si molds are summarized in Table S1 in the Supplementary Material [36,41,42,43,44,45,46,47,48]. The most important benefit of Si molds is that various micro/nanostructures can be fabricated via accessible cleanroom processing. Furthermore, the molds possess excellent surface quality in terms of roughness and flatness. However, despite the improvements in the fabrication techniques for Si molds, their use in glass imprinting is limited for two important reasons: (i) Si molds are oxidized at high temperatures, and (ii) a protective coating is required to avoid them adhering to the glass substrate. The thickness of this protective coating generally ranges from 30 to 130 nm. Moreover, the durability of Si molds deteriorates with repeated glass imprinting under high temperatures and pressures. These limiting factors confine the use of Si molds to the molding of ultra-low *T_g_* to low *T_g_* glass only.

### 2.2. Ni and Ni-Alloy Molds

**Figure 4:**
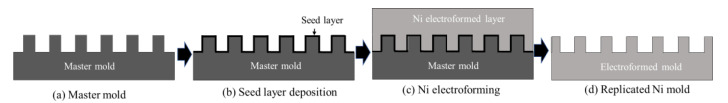
Schematic illustration of the fabrication of a Ni mold using electroforming: (**a**) master mold, (**b**) deposition of the conducting seed layer, (**c**) deposition of the Ni electroformed layer, and (**d**) removal of the Ni electroformed layer.

Transition metals such as nickel (Ni) and their alloys are frequently utilized as mold materials for polymer and glass replications [49,50,51] due to their hardness, ductility, corrosion resistance, and low thermal expansion coefficient. Depending on the desired pattern geometry and dimension, a Ni mold can be fabricated by electroforming on the master pattern or micromachining of a Ni alloy layer plated on other metal blocks such as steel [52]. Electroforming is usually preferable when the desired pattern dimension is typically sub-tens micron or submicron scale, which are difficult to obtain by precision machining. A schematic diagram of Ni mold fabrication via electroforming is presented in Figure 4. During electroforming, the Ni mold is replicated from a master mold in a chemical bath solution. For example, Hsu et al. [50] reported the fabrication of a Ni mold with V-microgroove patterns produced via electroforming. Then, these microgrooves were replicated on FCD1 and SF2 glass using glass isothermal molding.

Precision machining can provide high-dimensional accuracy and surface finish of complex geometry. In general, pure nickel or other Ni-based alloys are known as some of the most difficult-to-machine due to work hardening, tool wear, and the chemical reaction between the tool and workpiece. Fortunately, nickel–phosphorus (Ni–P) alloy is one exception as it is not difficult to machine, it can be applied to machine the Ni alloy-based mold [53,54]. Ni alloy mold can be fabricated by electro or electroless plating of Ni alloy on a support metal block followed by micromachining of Ni alloy layer to obtain the desired pattern. For instance, Yi et al. [55] reported the fabrication of a Ni alloy mold with a concave aspherical surface using an electroformed Ni alloy on a stainless steel substrate and ultraprecision diamond turning with the fast servo tool. The reported surface roughness of the machined aspherical profile on the Ni alloy mold was in the range of 15–25 nm.

Since conventional machining has a limitation in the machining of Ni alloy, other than for Ni–P alloy, laser machining can be applied for machining the other Ni alloy. For example, Chen et al. [56] utilized laser machining on Inconel 601 (Ni-Cr-Al alloy) for fabricating a mold for glass microfluidic devices. Then, the mold layout was transferred to a soda-lime glass substrate for DNA analysis. The polishing and cleaning as a post-process was conducted on the mold to remove the debris generated during laser machining on the mold. They observed that laser parameters, such as power, frequency, scanning speed, and scan number, greatly influenced the channel height and surface roughness. At optimized laser parameters, a maximum cutting depth of 125 µm was obtained with an average roughness of 4 µm.

Additional heat treatment can be applied to Ni–P alloy for enhanced mechanical and chemical properties. The electroformed Ni–P alloy has an amorphous crystalline structure. After electroforming, micromachining techniques are applied to produce the desired pattern on the mold surface. Additionally, heat treatment from 400 °C for 3 h to 600 °C for 1 h, which alters the physical and chemical properties without changing the shape, is conducted to increase the hardness of the mold. It has been observed that during thermal activation, transition from anamorphous phase to a meta-stable polycrystalline structure occurs, which enhances the mechanical strength [57,58]. As an example, Liu et al. [59] fabricated an array of microgrooves using ultraprecision diamond turning on a Ni–P alloy mold for glass molding. Upon heat treatment, the hardness of the Ni–P alloy mold was increased from 614 to 941 HV.

Adhesion between the mold and the glass is one of the most significant issues that can reduce the effectiveness of the glass molding process. To overcome this problem, Ni-tungsten (Ni–W) alloys have been proposed as a promising mold insert because Ni–W alloys have inherent release characteristics. It is believed that the release characteristics of the mold increase with the W content in the alloy. In mold fabrication, a low thermal-expansion alloy is commonly used as a dummy substrate for the deposition of Ni–W during electroforming. For example, Yasui et al. [60] reported a simple approach for Ni–W alloy mold fabrication using conventional lithography and electroforming. They used Incoloy909, a low coefficient of thermal expansion (CTE: 7.7 µm/m/°C) as a mold substrate. First, a 2 µm of Ni–W film was electroplated as a seed layer on the Incoloy909. Subsequently, periodic micropatterns were created using the SU8 negative photoresist on top of Ni–W film by photolithography. Then, the Ni–W pattern was electrodeposited through the SU8 pattern opening. The minimum pitch of the Ni–W pattern was 40 µm. In 23 repetitive glass-forming processes, the micro patterns were replicated on D263 borosilicate glass with good fidelity. They mentioned that the content ratio of W in the Ni–W affects the release characteristics of the mold. In their experiment, the W ratio in the Ni–W film was 28%. Yasui et al. [61] also introduced the Ni–W mold with nanogratings using electroforming followed by focused ion beam milling. They fabricated a line and space pattern with a length of 15 µm and width from 400 to 800 nm on the Ni–W mold surface. Then, the mold pattern was replicated on D263 borosilicate glass using hot embossing. As with Ni–P mold inserts, an amorphous Ni–W film can be crystallized by thermal annealing, resulting in higher mechanical properties and oxidation resistance. However, the heat treatment of Ni–W molds can be conducted immediately after micromachining or during hot embossing because the mold and glass are heated together to the target molding temperature.

Some examples of glass molding experiments that have employed Ni and Ni alloy molds are summarized in Table S2 in the Supplementary Material of S2 [50,55,56,57,58,60,61,62,63,64,65,66,67,68]. Ni and Ni alloy molds have high mechanical hardness, high corrosion resistance, and lower thermal expansion coefficients. A wide range of glass materials from ultra-low *T_g_* to low *T_g_* glass can be molded by exploiting Ni alloy mold inserts. Moreover, an anti-adhesion layer is not required for all Ni alloy molds because these molds have inherent release characteristics. In addition, various micro fabrication processes are available to fabricate Ni alloy molds. However, Ni alloy molds have some notable drawbacks. Ni alloy molds are mostly fabricated using the electrodeposition process. Inappropriate alloy composition or substrate material may lead to the electroformed layer peeling off completely due to internal stress. Therefore, the substrate material needs to be carefully selected, and the alloy composition needs to be optimized in advance. Moreover, the durability of Ni alloy molds deteriorates even after heat treatment when they are utilized at very high temperatures. During high-temperature molding, Ni atoms tend to diffuse from the alloy, resulting in a cloudy mold surface.

### 2.3. SiC Molds

SiC has been proposed as an alternative mold insert for the micro/nano hot embossing of glass materials. This is a Si-based ceramic material consisting of covalently bonded Si and carbon (C) in a crystalline lattice. There is a wide variety of stable SiC crystal structures, which are referred to as polytypes. Of these, 4H-SiC, 6H-SiC, and 3C-SiC are the most common due to their electrical, optical, and mechanical properties [69]. At high temperature, an oxide layer readily forms on the SiC surface, acting as a protective cover and leading to chemical inertness. SiC also offers low density, high hardness, good thermal conductivity, good wear resistance, and a low CTE.

**Figure 5:**
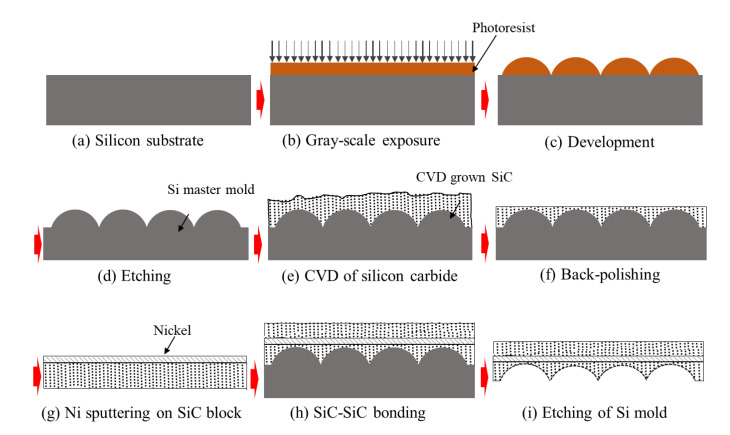
Schematic of SiC mold fabrication by Si lost molding: (**a**) Si substrate, (**b**) gray-scale lithography, (**c**) photoresist development, (**d**) etching, (**e**) deposition of SiC powder, (**f**) back polishing for smoothening, (**g**) sputtering of the Ni interlayer, (**h**) bonding with the SiC block, and (**i**) etching away of the Si substrate using a chemical solution.

Sintering is conventionally used to fabricate SiC molds, in which SiC powder is deposited on a pre-patterned substrate using chemical vapor deposition (CVD) followed by heating just below the melting temperature of SiC powder and, finally, by controlled pressing. During the sintering process, grains of SiC powder fuse together to form a dense and very hard SiC surface. After the sintering process, the SiC surface undergoes polishing and solid-state bonding with another SiC block with a Ni interlayer. A schematic of SiC mold fabrication using lost molding is shown in Figure 5. Min et al. [70] demonstrated an MLA on the SiC surface using lost molding. A SiC mold with the MLA was produced using grayscale lithography and RIE. Shin et al. [71] also studied an identical approach to producing a SiC stamp that had a line pattern with a width of 300 to 900 nm and square pits 250 to 900 nm wide. Then, these patterns were transferred to Pyrex glass through hot press molding. Another method for fabricating a SiC mold is reaction sintering, in which an amorphous SiC powder precursor (α-SiC 60 wt %, graphite 30 wt %, and phenolic resin 10 wt %) reacts with Si to produce a crystalline SiC structure [72] as follows [73]:

Si + C = SiC.(1)

In reaction sintering, a thin film of SiC is produced on a Si master mold using CVD. Next, SiC powder is placed on top of the initially formed SiC film. In the sintering of the SiC powder, SiC film is bonded with the newly generated SiC, resulting in dense SiC backing. As an example, Itohet al. demonstrated a SiC mold fabricated using reaction sintering with V-groove patterns [72]. Then, these V-groove patterns were transferred to Pyrex glass via glass molding. Unlike solid-state bonding, no interlayer is required in reaction sintering. However, the surface roughness produced via reaction sintering is higher than that from solid-state bonding [72] due to the mismatch between the newly produced and older SiC crystals.

In addition to sintering, SiC molds can be produced using conventional microfabrication and micromachining because SiC is a semiconducting material. For instance, Tamura et al. [74] developed an array of antireflection patterns with a pitch of 250 nm through electron beam writing and RIE. The absorption of UV laser light by SiC is fairly good, meaning that laser micromachining is another useful manufacturing approach. For example, Huang et al. [75] fabricated a microfabricated channel with a width of 200 µm and a depth of 185 µm on a sintered SiC surface using laser machining. However, laser machining the SiC surface produces a rougher surface compared to other approaches. The reported surface roughness (R_a_) with laser machining was 700 nm [75], compared to 1.2–8.0 nm for sintering [70,71,72]. However, the sintering process is more expensive than micromachining because the master mold is utilized only once. Another limitation of sintering is that polishing is required after SiC crystal deposition because SiC deposition produces an uneven surface. Moreover, the surface roughness of sintered SiC molds is associated with the deposition rate.

SiC is a sintered material that offers high hardness during molding experiments. Glass materials with an ultra-low to low *T_g_* can be molded easily using SiC mold inserts. The molding temperature for SiC molds ranges from 420 to 850 °C without any further processing. Table S3 in the Supplementary Material presents examples of micro/nano glass imprinting using SiC molds [70,71,72,74,75,76,77]. However, during molding trials, an anti-adhesion coating, with thickness from 45 to 1000 nm, is required to prevent the mold from sticking to the glass material. Although the anti-adhesion coating provides better imprinting and release characteristics, it requires additional processing steps, which subsequently increases the production costs.

### 2.4. WC Molds

Currently, one of the commonly used mold materials in industry is WC due to its exceptional hardness, toughness, and mechanical and wear resistance [78]. WC, generally known as a hard metal, is a composite material made primarily of W and C, which can also contain a metal binder such as cobalt (Co) to improve the mechanical properties (i.e., allowing for a tradeoff between hardness and toughness). Originally, WC was synthesized as powders with various mean particle sizes (micron, submicron, and nanometric), which strongly influences the physical properties of the WC alloy composite [79]. A very fine WC powder leads to higher stiffness and a smoother surface than do coarse particles with an identical composition.

Since WC is a very hard composite material, conventional lithographic and etching processes are rarely employed for the fabrication of WC mold inserts. Instead, direct micromachining techniques, such as precision grinding and diamond turning following sintering, are typically used to produce micro/nano features on WC mold surfaces. For example, Dambon et al. [80] used ultraprecision grinding to fabricate an aspherical surface on WC material. To minimize errors in form, finite element method (FEM) simulations were conducted before the precision grinding process. Huang et al. [81] also used diamond grinding to develop a Fresnel lens with three concentric rings with a teeth height of 50 to 220 µm. A platinum–iridium (Pt–Ir) alloy coating layer as a protective layer was applied to the mold before glass transcription. A notable difference in the surface finish can be observed when the WC surface is subject to different micromachining processes. In particular, ultraprecision grinding creates higher-quality mold inserts than does conventional grinding. For example, an ultra-smooth surface with a roughness of 5 nm [80] was achieved with ultraprecision grinding, while traditional grinding and polishing led to a roughness of 50 nm [82].

**Figure 6:**
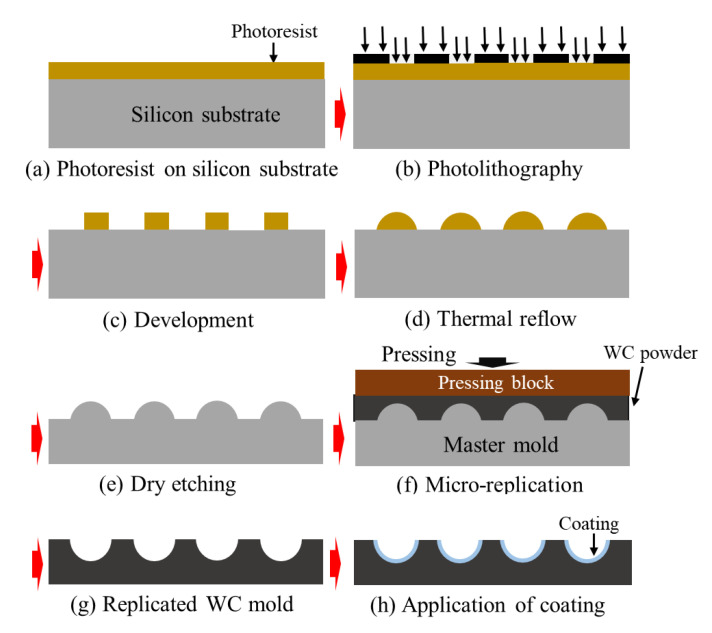
Schematic illustration of the fabrication of a WC mold using replication from a master mold: (**a**) photoresist layer on a Si substrate, (**b**) patterning photoresist layer, (**c**) development of the photoresist pattern, (**d**) thermal reflow, (**e**) dry etching of Si, (**f**) sintering of WC powder via replication, (**g**) replicated WC mold, and (**h**) anti-adhesion coating on the WC mold.

Another approach to fabricating WC mold inserts is replication via sintering from a master mold (Figure 6). First, a master mold with an MLA is produced using traditional thermal reflow methods. Then, the WC mold is replicated from the master mold via sintering. Han et al. [83] demonstrated the fabrication of a WC mold with an MLA pattern via replication from a Si master mold. An important advantage of this process is that the master mold can be used many times. However, significant shrinkage between the master mold and the replicated WC mold occurs during the sintering process. For instance, the diameter of the MLA patterns drops from 77 to 58 µm, the sag height decreases from 4.7 to 3.7 µm, and the period declines from 250 to 190 µm. Additionally, the surface roughness of the WC mold is more than 15 times that of the master mold. The large grain size of the WC powder may be responsible for this significant difference, with dislocations occurring during the sintering process.

A summary of previous reports of micro/nano glass molding with WC molds is presented in Table S4 in the Supplementary Material [22,80,81,82,83,84,85,86,87,88,89,90,91,92]. WC molds offer high hardness, good chemical resistance, and good thermal conductivity. The conventional methods for WC mold fabrication are grinding and polishing. For this reason, WC molds generally have a surface finish that is suitable for optical glass applications. A wide range of glass materials from ultra-low to high *T_g_* can be molded using a WC mold insert due to its thermal stability, with molding temperature ranging from 320 to 730 °C. However, a protective coating is required on the WC mold surface to avoid adhesion to the glass material. The thickness of this anti-adhesion coating ranges from 80 to 500 nm. In addition, there are few fabrication techniques available to manufacture WC molds due to its exceptional hardness. As such, diamond turning and grinding and polishing methods are typically used for the fabrication of geometric features on the WC surface.

### 2.5. GC Molds

GC (also known as vitreous carbon (VC) or amorphous carbon) has also been used as a mold material for the micro/nano molding of Al and glass materials [93,94,95]. Thermosetting organic polymers, such as phenol-formaldehyde, furfuryl alcohol, bisphenol, and acrylate, can be effectively converted to GC following heat treatment (1000–3000 °C) in an inert ambient atmosphere [96,97,98,99,100,101]. The resulting GC is chemically and thermally stable and has a low porosity, entailing impermeability to gases. It is also extremely hard, with a hardness of 6 to 7 on the Mohs scale, and it has a low CTE of 2.0 × 10^−6^ K^−1^ to 3.4 × 10^−6^ K^−1^ [101,102,103] at imprinting temperatures. Various methods, such as direct machining, traditional lithography, and the carbonization of a pre-patterned precursor, have been utilized to fabricate GC molds with micro/nano patterns. Laser machining, FIB, and sawing/dicing are representative examples of direct, mask-free machining techniques used to produce micro/nano features on the GC surface. In laser machining, micro-features on the GC surface are produced using a collimated light beam with a very high thermal energy. For example, Kuhnke et al. [104] employed a Nd:YAG diode laser to fabricate a micro-cell pattern on the surface of GC. Then, the micron-sized cell structure was transferred from the GC to soda-lime glass via glass molding. Tseng et al. [23] investigated a UV pulsed laser for the fabrication of a microchannel on the surface of GC for glass imprinting. Even though GC is a very hard material, laser machining can be utilized for the rapid production of a micropattern on a GC substrate. However, this approach has limitations in terms of the minimum feature size and surface roughness. The inability to produce nanoscale features is related to the diffraction limit of the laser beam, while the high surface roughness is due to debris from the evaporated material remaining on the surface as a result of the high thermal energy. However, the surface quality can be improved by adjusting the scanning speed of the laser beam. For example, the surface roughness falls from 672 to 80 nm when the scanning speed is reduced from 200 to 20 mm/s [23,105]. Therefore, to improve the surface quality, the laser scanning rate should be optimized for the specific application.

As with laser machining, dicing/sawing is also a fast machining technique for the production of micron-sized features on GC molds. Sawing is a low-cost, simple, flexible, and straightforward technique for fabricating patterns on GC surfaces. Various microstructures, such as micro cubes [106,107], micro pyramids [105,107], and line and space patterns [108], have been produced on the surface of GC using dicing. Then, these patterns were transcribed to glass materials via glass molding. However, sawing is also limited in terms of the minimum feature size and surface roughness of the resulting mold.

Another micromachining technique is FIB milling, in which material is removed by accelerating an ion beam toward the GC surface. For example, Takahashi et al. [109] fabricated line and space patterns from 2 µm down to 100 nm via FIB milling of a GC mold for glass imprinting. Takagi et al. [110] also reported the use of FIB milling to produce a GC mold with line and space patterns (width: 5–300 nm; depth: 150 nm) for glass imprinting. Unlike laser machining and dicing, FIB micromachining can produce micro/nano features with excellent surface quality. For example, a surface roughness of 5 nm with a machining height of 7.33 µm was reported for a GC surface using FIB milling [111]. However, the material removal rate for FIB milling is very slow in comparison to laser machining and sawing, which means it may not be suitable for scalable fabrication. Although a higher milling rate can be obtained by increasing the beam current, this increases the surface roughness. Another constraint is the redeposition of etchant residues (i.e., ions), which contaminate the etched profile of the mold. To remove the redeposited ions, heat treatment in an atmospheric or vacuum environment at an elevated temperature (up to 1400 °C) for several minutes is useful. Nevertheless, this removal of etchant ions decreases the surface quality, with atmospheric heat treatment more damaging in this respect than vacuum heat treatment [109]. To take advantage of the speed of laser machining and the good surface quality of FIB milling, Youn et al. [105] proposed a combination of laser ablation and FIB milling to fabricate GC molds and observed that the mold surface quality improved almost two-fold, with the roughness falling from 45 from 80 nm.

Conventional micro/nanofabrication has also been exploited for the production of micro/nano-sized structures on GC molds. For instance, Chen et al. [112] used traditional photolithography and RIE to develop microholes with 100 µm diameter and 10 µm height on the surface of GC. Then, these microholes were imprinted on glass followed by the transformation into an MLA using the thermal reflow of the glass. Yasui et al. [113] proposed combining electron beam writing with plasma-coupled RIE to fabricate line and space patterns with a depth of 300 nm on the surface of GC. Then, these grating patterns were transcribed to Pyrex glass using hot embossing. The advantage of standard lithography and etching is that micron to submicron structures can be produced with vertical sidewalls. Moreover, the fabricated patterns have high surface quality. For instance, a surface roughness of 3 nm can be achieved when fabricating patterns onto GC surfaces [114]. However, etching on a GC surface is challenging because GC is very hard and chemically stable. Mekaru et al. [114] reported an etch rate of 1.8 nm/s with oxygen and sulfur hexafluoride plasma, which was attributed to the fact that the dry etching of GC material is very slow. Moreover, solution-based chemical etching is rarely utilized for GC mold fabrication because GC exhibits isotropic etching behavior due to its amorphous structure [115].

**Figure 7:**
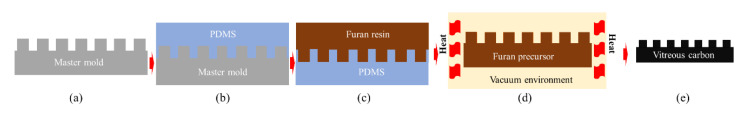
Schematic illustration of the fabrication of a GC mold: (**a**) master mold, (**b**) polydimethylsiloxane (PDMS) casting, (**c**) furan casting, (**d**) carbonization in a vacuum or inert ambient environment, and (**e**) the GC mold.

Another technique used to fabricate GC molds is the carbonization (also called pyrolysis) of pre-patterned polymers. Micro/nano patterning on the polymer material can be carried out in two ways: (i) replication from the master pattern and (ii) patterning via lithographic approaches. After patterning the polymer precursor, carbonization is carried out in an inert environment to obtain the GC mold. A schematic of the GC mold fabrication is shown in Figure 7. As an example, Ju et al. [116] obtained a GC mold with nano grating patterns for glass replication via the carbonization of a furan precursor. Kim et al. [117] also reported a GC mold with a Fresnel lens for glass molding via the carbonization of a furan polymer. The main advantage of this technique is the low fabrication costs because the same master can be used repeatedly, and inexpensive polymer precursor materials are available. In addition, a cleanroom environment is not required, and the surface roughness of the fabricated mold remains very close to that of the master pattern. For instance, the reported root mean square (RMS) values for the surface roughness of the master and GC molds were 2.17 nm and 4.78 nm, respectively [117]. However, dimensional shrinkage of the micro/nanostructures occurs during the thermal decomposition of polymer materials. This anisotropic shrinkage depends on the C content of the precursor resins [118]. Although this reduction in size can be beneficial for the conversion from micro to nano patterns, a master mold with a larger pattern needs to be designed to obtain a GC mold with the desired pattern geometry.

Examples of micro/nano glass molding using GC molds are summarized in Table S5 of the Supplementary Material [23,105,106,107,108,109,110,111,112,113,114,116,117,119,120,121,122,123,124,125,126,127,128,129,130]. There are approaches available to fabricate GC molds. They have good thermal conductivity, which enables large-area micro/nano glass molding. Almost all optical glass materials, from ultra-low to very high *T_g_* glass, can be molded using a GC mold insert. The molding temperature for GC molds ranges from 380 to 1360 °C, while no anti-sticking coating is required due to its C content. However, due to its exceptional hardness and chemical stability, the material removal rates are very low in the dry etching process, which increases the manufacturing costs. Thus, carbonization (or pyrolysis) of the predefined polymer can be a good candidate for the low-cost and scalable fabrication of VC mold. In this case, it is very important to predict the shrinkage of the pattern during the carbonization and reflect it in the master fabrication.

### 2.6. Other Mold Insert Materials

Cemented carbide (CC), Al alloys, polycrystalline diamond, and fused silica have also been used as mold inserts in micro/nano glass molding. CC is a composite material typically used for cutting and wearing tools. Three forms of CC are available: (i) WC-based CC with a Co binder; (ii) WC, titanium carbide (TiC), tantalum carbide (TaC), and niobium carbide (NbC)-based CC with a Co binder; and (iii) TiC-based CC with a Ni binder, referred to as a cermet (Ti (C,N)–Mo_2_C–WC–Ni) [131]. Powdered metal particles are bonded through sintering or hot isotropic pressing in the presence of a Co or Ni binder to produce CC. The mechanical properties (e.g., hardness, ductility, and wear resistance) of CC can be controlled by adjusting the composition, binder, and process parameters. To fabricate a mold insert using CC, micromachining is a suitable approach to ensure geometric accuracy and surface smoothness. For example, Zhu et al. [132] performed ultraprecision diamond grinding on CC CW500 followed by the addition of a diamond-like carbon (DLC) coating to produce positive and negative aspherical surfaces. Then, these aspherical surfaces were transferred to chalcogenide glass to fabricate a double-sided aspherical lens for an IR detector. However, shape errors and high surface roughness were introduced during the diamond grinding process. To overcome this, the ground carbide mold underwent further machining and polishing to attain the expected shape precision and surface roughness.

Al alloy 6061, containing Si, magnesium, and Cu as the main alloy elements [133], can also be used as a mold insert for glass molding. As with CC, direct machining is a feasible approach to the production of micron-sized features with high accuracy and good surface quality. Ultraprecision diamond turning integrated with slow tool servo computer numeric control (STS-CNC) has been used to create an MLA on the surface of Al alloy 6061 with high accuracy and good surface quality [134]. The observed surface roughness for the mold with STS-CNC was 13.65 nm, which is appropriate for optical applications. Then, a glass molding experiment was conducted to transcribe the MLA patterns from the Al alloy mold to IR glass. Zhang et al. also observed Al 6061 alloy mold with a microlens array with an optical surface finish for chalcogenide glass molding for infrared imaging applications [135].

Another mold insert technique involves the use of a polycrystalline diamond mold, which can be fabricated using FIB milling because CVD diamond is a very hard material (10,000 HV) [136]. Komori et al. created micro pits and various line and space patterns using FIB milling on a CVD diamond mold with exceptional surface quality. Then, a glass replication test was conducted, and the micropatterns were successfully transferred to various glass materials such as Pyrex, Tempax, and BK7.

The difference in the CTE of bare Si and silicon dioxide (SiO_2_) is 2.9 × 10^−6^/°C and 5.8 × 10^−6^/°C at room temperature and 900 °C, respectively [137]. To cope with the rapid change in temperature during molding experiments, a thin layer of SiO_2_ on a Si-based mold or fused silica mold leads to a better CTE than does bare Si. For instance, Hirai et al. [138] deposited a SiO_2_ layer on a Si substrate followed by deep UV light lithography and RIE to form line and space patterns with widths of 250 nm to 1.0 µm. Then, these subwavelength grating structures were transferred from the silica mold to a glass substrate using glass molding. Li et al. [139] reported the fabrication of a fused silica mold with a square patterned array with an area of 10 µm^2^ and a height of 0.8 µm. The patterned mold was fabricated using a traditional lithography and dry etching process and coated with a thin layer of graphene to prevent adhesion during the release process. Then, these patterns were transcribed to glass blanks using glass molding.

Examples of micro/nano glass molding with CC, Al alloys, polycrystalline diamond, and fused silica molds are summarized in Table S6 in the Supplementary Material [132,134,135,136,138,139,140,141,142,143]. CC is a very hard alloy material that can be utilized for the molding of both low and high *T_g_* glass material. However, due to its high hardness, direct machining is the only means to produce the desired geometric features on the mold surface. Moreover, anti-adhesion coating is required to improve the demolding process. Al alloys have also been employed for the fabrication of mold inserts for ultra-low *T_g_* glass. However, at elevated temperatures, their mechanical strength decreases due to the intermetallic alloy composition. For this reason, Al alloy molds are not suitable for the molding of high *T_g_* glass. CVD-grown diamond molds have high hardness and offer high temperature conduction, which makes them suitable for molding a wide range of glass materials. In addition, no coating is required for the molding process, even for high *T_g_* glass, due to its intrinsic C content. Despite this, CVD-grown diamond molds are inherently polycrystalline in nature, which can increase the surface roughness during the machining process. In addition, it is very difficult to produce geometric features on the mold surface due to its exceptional hardness. For this reason, only a limited range of micromachining approaches are suitable for the machining of the surface of a diamond mold. Fused silica or SiO_2_ molds are also suitable for the molding of low *T_g_* glass materials. Conventional lithography and etching can be employed for the manufacturing of these mold inserts. Moreover, it is possible to manufacture very small nanometer-scale features on the mold surface with excellent flatness and surface smoothness. Nevertheless, fused silica is not an effective mold material due to its adhesion properties at high temperatures. At elevated temperatures, a chemical reaction occurs between the glass and silica mold, which leads to sticking behavior. Furthermore, dummy molds are required during photolithography because silicate materials are transparent in the visible spectrum.

## 3. Discussion

**Figure 8:**
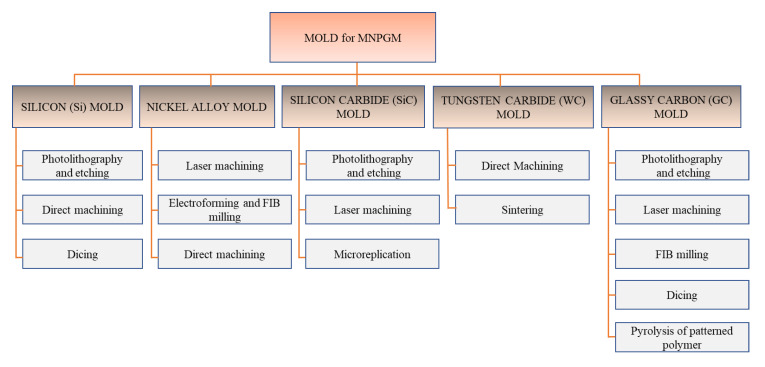
Schematic representation of the various mold fabrication techniques available for precision glass molding.

Mold inserts can be fabricated in a number of ways to achieve the desired surface texture with a smooth surface finish and form accuracy. To develop commercially viable glass molding technology, fabrication approaches should be easy and inexpensive, because the design and fabrication processes directly influence the cost of the final product. A taxonomy of mold fabrication approaches is presented in Figure 8 based on the information presented in the papers summarized in Tables S1–S6. Depending on the selected mold material, different fabrication methods are available to produce the target patterns. A fabrication method such as photolithography may be practical for fabricating a pattern on one mold surface (e.g., Si) but impractical for another (e.g., Ni alloy). Conventional microfabrication techniques (lithography and etching) offer design flexibility for the production of 2D miniature patterns and very smooth surface profiles. However, these methods are expensive and only suitable for certain materials. Furthermore, they require a complex mask design, the use of harmful chemicals, many processing steps with various constraints, regular maintenance, and a cleanroom environment.

In direct machining, 3D microstructures with ultra-low surface roughness (e.g., 2 to 80 nm) can be produced without masking, but the pattern geometry is limited by the tool dimensions. To fabricate a desired pattern, a unique diamond tool with a suitable angular orientation is required, and it cannot be reused for other patterns. Additionally, during the micromachining process, the surface energy of the removed material (referred to as the chip) and workpiece rises, resulting in chip adherence to the workpiece/substrate, which subsequently increases the surface irregularities. To facilitate chip removal during machining, an appropriate cutting fluid should be employed in the cutting direction. Machining with the assistance of a cutting fluid is referred to as wet cutting, while dry cutting does not use a cutting fluid. In contrast, a wide range of microstructures can be fabricated in a relatively short time using laser machining (another mask-less approach) simply by adjusting the laser parameters (e.g., the power of the laser, the scanning speed, and duration of laser exposure). Laser machining is also useful for a wide variety of materials, such as metals, ceramics, and polymers. However, the surface roughness in laser machining is relatively high due to the diffraction limit of laser light and the redeposition of vaporized material. Furthermore, laser machining is material-dependent, and microcracks may form in heat-affected zones.

As with direct machining, 3D structures with a high aspect ratio and fine surface finishing can be obtained using FIB milling. For instance, a surface roughness of 5 to 20 nm was observed using FIB milling. However, FIB milling is impractical for large-area patterns due to its very slow milling rate. Similarly, a very limited range of microstructures with a comparatively high surface roughness can be obtained using the dicing process due to its sequential machining process. The lowest surface roughness value obtained with dicing was 150 nm. Flexible microstructures with excellent surface quality can be obtained via electroforming and sintering by adjusting the processing parameters, such as the deposition rate, sintering temperature, and sintering pressure. For less expensive micro/nano pattern fabrication on the surface of a mold, the carbonization of patterned polymers is a good choice, but dimensional shrinkage is an issue that must be compensated for in order to produce the desired pattern. The amount of shrinkage depends on the carbon content of the precursor material: the minimum was found to be 22% from master to glass product.

**Figure 9:**
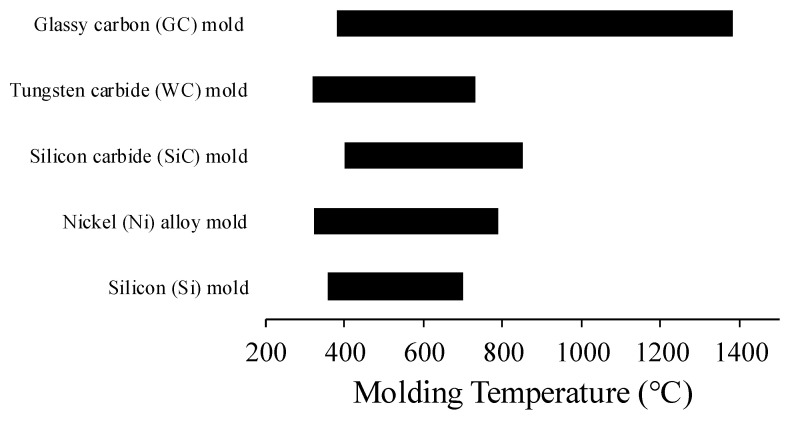
Comparison of molding temperatures for different mold types.

A crucial feature of mold inserts is their thermal stability during the molding process for a wide range of temperatures (i.e., for ultra-low to very high *T_g_* glass molding). A comparison of the molding temperatures of the various types of mold insert is presented in Figure 9 based on the data from the papers summarized in Tables S1–S6. Every type of mold insert can be used for ultra-low to low *T_g_* glass molding. However, a coating is sometimes required during molding at high temperatures to avoid adhesion between the mold and glass, except with GC molds, which can be used even for ultra-high *T_g_* glass molding (e.g., quartz glass molding, *T_g_* = 1200 °C) without an anti-sticking layer. Although it is commonly believed that a hard coating on the mold surface increases the durability of the mold and reduces the cleaning and maintenance costs, the coating process requires additional processing steps that increase the operating costs. In addition, there are two important considerations when applying a coating layer: (i) the CTE of the mold material and the coating, which should be similar, and (ii) the uniformity of the coating thickness. Otherwise, the possibility of the coating peeling off the mold surface with repeated molding cycles is high.

## 4. Future Outlook

In micro/nano glass molding, the quality of the molded glass products is directly influenced by the quality of molds. Currently, various mold insert techniques are available, which are being used for the molding of glass material with excellent efficiency. However, there remain shortcoming with the existing mold inserts technologies, such as intricate and expensive manufacturing, requirements of coating, and discharging of coating materials during repeated molding at high temperature and pressure. To make the micro/nano glass molding technology commercially viable, easy, and cost effective, micro/nano mold fabrication techniques should be proposed. Additionally, coating is used to avoid the sticking tendency and increase molding trials, so coating technologies could be further improved for better performance. In addition, it is well known that if the mold material inherently contains a carbon substance, the coating requirement may be avoided. Hence, new technology could be developed to fabricate carbon alloy molds.

## 5. Conclusions

This review discussed the fabrication of commonly used mold inserts for micro/nano glass molding. There are various mold insert techniques involving the use of Si, Ni alloys, SiC, WC, GC, and other mold materials. A mold can be manufactured using a wide variety of techniques depending on the properties of the mold material. Despite the unique advantages of each technique, there are some obvious limitations as well. In addition, because micro/nano glass structures are mostly used for light manipulation, the surface finishing of the mold should be compatible with optical applications. Although the commercial viability of existing mold fabrication techniques has already been proven, there remains room for further development. Therefore, we believe that this review is beneficial for research groups who are aiming to develop optimal mold manufacturing processes for the production of desired micro/nanostructures on glass substrates using glass molding.

## Supplementary Materials

The following are available online at https://www.mdpi.com/article/10.3390/mi12070812/s1, Table S1: Summary of studies on micro/nano glass molding using Si molds, Table S2: Summary of studies on micro/nano glass molding with Ni-alloy molds, Table S3: Summary of studies on micro/nano glass molding with SiC molds, Table S4: Examples of studies on micro/nano glass molding with WC molds, Table S5: Summary of studies of GC micro/nano mold insert systems for glass imprinting, Table S6: Summary of studies on other micro/nano mold insert systems for glass imprinting.
