# Supplementary material for: A Comprehensive Review of Micro/Nano Precision Glass Molding Molds and Their Fabrication Methods
Source: Micromachines (Basel). 2021 Jul 12;12(7):812. doi: 10.3390/mi12070812 (PMC8304585; doi:10.3390/mi12070812)
Supplement: Supplementary file 1 [file micromachines-12-00812-s001.zip › micromachines-1259209-supplementary.pdf]

[Supplementary Materials]

Table S1: Summary of studies on micro/nano glass molding using Si molds. **Acronyms:** CBG: carbon-bonded graphene; RIE: reactive ion etching; EBL: electron-beam lithography; MLA: microlens array; OLED: organic light-emitting diode; Si_3N_4: silicon nitride; KrF: krypton fluoride; N/A: not available.

Authors & Affiliations	Mold fabrication methods	Pattern geometries on the mold	Glass materials	Anti-adhesion coating materials	Molding conditions	Molding methods	Mold surface roughness (R_a)	Proposed/ tested applications
Saotome et al./Gunma University, Japan[36]	EBL and anisotropic etching	V-grooves with throat widths ranging from 100 nm to 2.0 μm	K-PSK 100<br>T_g: 390°C<br><br>K-PG 375<br>T_g: 344°C	NA	Temperature: 357 °C<br>Pressure: 10 MPa	Isothermal molding	5 nm	V-groove microstructure
Li et al./Hong Kong Polytechnic University, Hong Kong[41]	Precision diamond machining	MLA<br>Size (period): 60 μm<br>Sag height: 900 nm	P-SK75<br>T_g: 493°C	CBG coating<br>Thickness: 130 nm	Temperature: 700°C<br>Pressure: Direct pressing	Active heating	2 nm	Light manipulation

Chen et al./Ohio State University, USA[42]	Ultraprecision diamond turning and RIE	Fresnel lens	K-PG325<br>T_g: 285°C	CBG coating	N/A	Isothermal molding	88 nm	Optical applications
He et al./Ohio State University, USA[43]	Photolithography and plasma-assisted RIE<br><br>Precision diamond turning and RIE	Microwells with a width of 11μm and a height of 1.5 μm<br><br>Fresnel lens with a diameter of 9 mm and a teeth depth of 1μm	P-LASF47<br>T_g: 530°C	CBG coating<br>Thickness: 45 nm	Temperature: 640 °C<br>Pressure: 0.22 MPa	Active heating	0.5 nm	Image sensing
Hung et al./National Tsing Hua University, Taiwan[44]	Dicing followed by traditional lithography and wet etching	Line and spacing patterns with a width of 20 μm and a depth of 20 μm, 40 μm, and 200 μm	K-PG375<br>T_g: 344°C	Si_3N_4 coating<br>Thickness: 50 nm	Temperature: 352°C<br>Pressure: 2.1 MPa	Isothermal molding	N/A	Optical applications

He et al./Ohio State University, USA[45]	Precision diamond turning	Kinoform (Fresnel) lens<br><br>MLA dimensions: 360 μm ×360 μm<br>Sag height: 4.6 μm	P-SK57<br>T_g: 493°C	CBG coating	Temperature: 560°C<br>Pressure: 20 kPa	Isothermal molding	15 nm	Light manipulation
Zawawi et al./Yonsei University, Korea, Republic of[46]	KrF lithography and RIE	Nanograting structure with a width of 500 nm and a depth of 335 nm	K-PG375<br>T_g: 344°C	Si_3N_4 coating<br>Thickness: 30 nm	Temperature: 370.9 °C<br>Pressure: 0.2 MPa<br><br>Temperature: 360°C<br>Pressure: 2.3 MPa	Active heating<br><br>Isothermal molding	3 nm	Optical applications
Ishihara et al./Kyoto University, Japan[47]	EBL and plasma-assisted RIE	Nanograting pattern with a pitch of 300 nm, a diameter of 200 nm, and heights ranging from 40 nm to 60 nm	K-PG375<br>T_g: 343°C	N/A	Temperature: 360°C<br>Pressure: 2.2 MPa	Isothermal molding	N/A	OLED applications

Li et al./Hong Kong Polytechnic University, Hong Kong[48]	Single-point diamond machining	MLA<br>Size (period): 60 μm Sag height: 900 nm	P-SK75<br>T_g: 493°C	CBG coating<br>Thickness: 60 nm	Temperature: 565°C<br>Pressure: 43.5 kPa	Active heating	6.2 nm<br>to 10.75 nm	Optical applications

Table S2: Summary of studies on micro/nano glass molding with Ni-alloy molds. **Acronyms:** FIB: focused ion beam; DLC: diamond-like carbon; MLA: microlens array; N/A: not available.

Authors & Affiliations	Mold fabrication methods	Pattern geometries on the mold	Glass materials	Anti-adhesion coating materials	Molding conditions	Molding methods	Mold surface roughness (R_a)	Proposed/ tested applications
Hsu et al./National Chiao Tung University, Taiwan[50]	Electroforming	V-groove patterns<br>Width:50μm<br>Height: 25 μm	FCD1<br>T_g: 455°C<br>SF2<br>T_g: 430°C	Boron nitride	Temperature: 490°C<br>Pressure: 15 MPa<br>Temperature: 485°C<br>Pressure: 15 MPa	Isothermal molding	N/A	Optical communication
Yi et al./Ohio State University, USA [55]	Fast servo-assisted ultraprecision diamond machining	Aspherical lens	K-PG325<br>T_g: 285°C	N/A	Temperature: 325°C<br>Pressure: 3.18 MPa	Isothermal molding	14.39 nm	Imaging applications
Chen et al./Polytechnic of Turin, Italy [56]	Laser micromachining	Microchannel<br>Width: 500 μm	Soda-lime glass<br>T_g: 580°C	NA	Temperature: 680°C<br>Pressure: 2.4 kPa	Isothermal molding	4 μm	Microfluidic applications

		Height: 160 µm						
Mekaru et al./National Institute of AIST, Japan [57]	FIB milling	MLA with a sag height of 4.5 µm and a radius of 12 µm and 20 µm<br><br>AIST logo with a character width of 10 µm or less	Pyrex glass<br><br>T_g: 560°C	N/A	Temperature: 640°C<br><br>Pressure: 2.5 MPa	Isothermal molding	N/A	Light manipulation
Zhou et al./Beijing Institute of Technology, China [58]	Single-point diamond turning	Microgroove pattern<br><br>Period: 10 µm<br><br>Height: 5 µm	KPG-325<br><br>T_g: 288°C<br><br>PG-375<br><br>T_g: 344°C	N/A	Temperature: 330°C<br><br>Pressure: 10 MPa<br><br>Temperature: 380°C<br><br>Pressure: 10 MPa	Isothermal molding	N/A	Electronic displays

Yasui et al./Kanagawa Industrial Technology Center, Japan [60]	Electroforming and photolithography	Micrograting patterns<br><br>Period: 40 μm<br><br>Mean depth: 4.15 μm	Borosilicate glass (D263)<br><br>T_g: 557°C	No coating	Temperature: 610°C<br><br>Pressure: 0.89 MPa	Isothermal molding	NA	Micro-optical applications
Yasui et al./Kanagawa Industrial Technology Center, Japan [61]	FIB milling	Line and space pattern<br><br>Width: 400 nm to 800 nm<br><br>Length: 15 μm<br><br>Square pit<br><br>Size: 20 μm × 20 μm<br><br>Height: 6.5 μm	Borosilicate glass (D263)<br><br>T_g: 557°C	No coating	Temperature: 650°C<br><br>Pressure: 6.37 MPa<br><br>Temperature: 590°C<br><br>Pressure: 0.66 MPa	Isothermal molding	20 nm to 40 nm	Microfluidic applications
Mekaru et al./National Institute of AIST, Japan [62]	FIB milling	MLA<br><br>Height: 4.5 μm<br><br>Radius: 12 μm and 20 μm	Pyrex glass<br><br>T_g: 560°C	N/A	Temperature: 620°C<br><br>Pressure: 2.5 MPa	Isothermal molding	N/A	Optical applications

Chen et al./Polytechnic of Turin, Italy [63]	Laser micromachining	Microchannel<br>Width: 500 μm<br>Height: 160 μm	Soda lime glass,<br>T_g: 580°C<br><br>Pyrex glass<br>T_g: 640°C<br><br>Magneto-optical glass<br>T_g: 360°C	N/A	Temperature: 660°C<br>Pressure: 2.407 kPa<br><br>Temperature: 710°C<br>Pressure: 2.1 kPa<br><br>Temperature: 410°C<br>Pressure: 1.929 kPa	Furnace heating-assisted molding	4 μm	Microfluidic applications
Mekaru et al./National Institute of Advanced Industrial Science and Technology (AIST), Japan [64]	FIB milling	Line and space patterns with linewidths of 500 nm, 750 nm, and 1000 nm and a depth of 3 μm<br><br>Dot arrays	Pyrex glass<br>T_g: 560°C	N/A	Temperature: 600°C<br>Pressure: 63.69 kPa	Isothermal molding	N/A	Microfluidic applications

Zhou et al./Tohoku University, Japan [65]	Single-point diamond turning	V-groove patterns<br>Width: 10 μm<br>Height: 5 μm	L-BAL42<br>T_g: 506°C	N/A	Temperature: 570°C<br>Pressure: 2.72 MPa	Isothermal molding	2 nm	Biomedical applications
Katsuki et al./Advanced Optical Manufacturing Technologies, Japan [66]	Diamond turning	MLA<br>Sag height: 6.56 μm<br>Aperture: 39.78 μm	PG-375<br>T_g: 344°C	N/A	Temperature: 375°C<br>Pressure: 7 MPa	Isothermal molding	N/A	Mobile phone cameras
Kim et al./Yonsei University, Korea, Republic of [67]	Electroforming	MLA<br>Diameter: 80 μm<br>Pitch: 100 μm<br>Sag height: 15.2 μm	K-PSFn214<br>T_g: 425°C	DLC coating: 500 nm	Temperature: 790°C<br>Pressure: 1 MPa	Active heating	1 nm	Optical imaging
Chen et al./Politecnico di Torino, Italy [68]	Laser machining followed by polishing	Microchannel<br>Dimensions: 140μm× 150 μm	Soda-lime glass<br>T_g: 564°C	N/A	Temperature: 640°C<br>Pressure: 7 kPa	Isothermal molding	40 nm	DNA analysis

Table S3: Summary of studies on micro/nano glass molding with SiC molds. Acronyms: UV: ultraviolet; Si: silicon; EBL: electron-beam lithography; RIE: reactive ion etching; CBG: carbon-bonded graphene; C-film: carbon film; GC: glassy carbon; DLC: diamond-like carbon; N/A: not available.

Authors & Affiliations	Mold fabrication methods	Pattern geometries on the glass	Glass materials	Anti-adhesion coating materials	Molding conditions	Molding methods	Mold surface roughness (R_a)	Proposed/ tested applications
Minet al./Tohoku University [70]	Replication from Si master mold	Non-spherical lens<br>Diameter: 300 μm<br>Height: 14 μm<br><br>Trench and pits:<br>Width: 5–20 μm	K-PG395<br>T_g: 363°C	DLC coating<br>Thickness: 1000 nm	Temperature: 400°C<br>Pressure: 2.45 MPa	Isothermal molding	1.2 nm	Optical communication
Shin et al./Tohoku University [71]	Replication from Si master mold	Nanograting patterns<br>Width: 300 nm<br>Pitch: 600 nm	Pyrex<br>T_g: 560°C	No coating layer	Temperature: 800°C<br>Pressure: 1 MPa	Isothermal molding	N/A	Optical applications
Itoh et al./Tohoku University, Japan [72]	Replication by reaction bonding from Si master mold	Triangular structures<br>Pitch: 5–20 μm	Pyrex<br>T_g: 560°C	GC coating	Temperature: 850°C<br>Pressure: 1 MPa	Isothermal molding	8 nm	Optical fiber networks
Tamura et al./Hokkaido University [74]	EBL and RIE	Antireflection structures<br>Pitch: 250 nm	K-PSK100<br>T_g: 390°C	C-film coating<br>Thickness: 50 nm	Temperature: 420°C<br>Pressure: 5 MPa	Isothermal molding	N/A	Antireflection

Huang et al./National Chiao Tung University, Taiwan [75]	UV laser micromachining	Microchannel Width: 200 μm Depth: 185 μm	Soda-lime glass T_g: 573°C	CBG coating Thickness: 45 nm	Temperature: 620°C Pressure: 50 kPa	Isothermal molding	700 nm	Bioanalysis
Yamada et al./National Institute of AIST[76]	EBL and plasma-assisted RIE	Antireflection structures Depth: 250 nm and 290 nm Pitch: 300 nm	K-PSK100 T_g: 390°C	C-film coating Thickness: 50 nm	Temperature: 430°C Pressure: 5 MPa	Isothermal molding	N/A	Antireflection
Huang et al./National Applied Research Laboratories, Hsinchu, Taiwan[77]	UV laser micromachining	MLA Diameter: 20 mm Sag height: 52 μm Pitch: 700 μm	Soda-lime glass T_g: 573°C	N/A	Temperature: 630°C Pressure: 6.6 to 9.1 MPa	Isothermal molding	N/A	Uniform light illumination

Table S4: Examples of studies on micro/nano glass molding with WC molds. Acronyms: MLA: microlens array; DLC: diamond-like carbon; Pt: platinum; Pt–Ir: platinum-iridium; Re-Ir: rhenium-iridium; N/A: not available.

Authors & Affiliations	Mold fabrication methods	Pattern geometries on the glass	Glass materials	Anti-adhesion coating materials	Molding conditions	Molding methods	Mold surface roughness (R_a)	Proposed/ tested applications
Cha et al./Korea Photonics Technology Institute, Korea [22]	Grinding and polishing	Aspherical lens	L-BSL7 T_g: 498°C	DLC coating Thickness: 80 nm	Temperature: 555°C Pressure: 50 kPa	Isothermal molding	4.018 nm	Mobile phone cameras
Dambon et al./Fraunhofer Institute for Production Technology, Germany [80]	Ultraprecision grinding	Aspherical lens Diameter: 22 mm	P-SK 57 T_g: 493°C	N/A	Temperature: 545°C Pressure: 7.9 MPa	Isothermal molding	less than 5 nm	Optical imaging
Huang et al./Hsinchu Science Park, Taiwan [81]	Precision diamond grinding	Fresnel lens	K-CSK120 T_g: 489°C	Pt–Ir alloy coated	Temperature: 560°C Pressure: 1.67 MPa	Isothermal molding	N/A	CPV applications

Aono et al./Nikon Corporation, Japan[82]	Precision grinding and polishing	Aspherical lens<br>Diameter: 60.5 mm	SK5<br>T_g: 667°C	N/A	Temperature: 720–730°C<br>Pressure: 0.63MPa	Isothermal molding	10 nm	Optical imaging
Han et al./ Yonsei University, Korea [83]	Replication by sintering from a master mold	MLA<br>Sag height: 3.6 μm<br>Period: 190 μm	K-PSK100<br>T_g: 390°C	Pt coating	Temperature: 415°C<br>Pressure: 1.27 kPa	Isothermal molding	less than 53 nm	Laser beam focusing
Chen et al./Hsinchu Science Park, Taiwan[84]	Ultraprecision diamond grinding	MLA<br>Sag height: 8.5 μm	K-CSK120<br>T_g: 498°C	N/A	Temperature: 550–570°C<br>Pressure: 15 kPa	Isothermal molding	10 nm	Light intensity modulation
Kim et al./Korea Photonics Technology Institute, Korea [85]	Ultraprecision grinding and polishing	Aspherical lens	Chalcogenide glass NBU-IR1 ($Ge_{20}Sb_{15}Se_{65}$)<br>T_g: 285°C	DLC coating<br>Thickness: 100 nm	Temperature: 320°C<br>Direct pressure	Isothermal molding	17.5 nm	Cameras for night vision
Kim et al./Korea Photonics Technology Institute, Korea [86]	Ultraprecision grinding and polishing	Aspherical lens	Chalcogenide glass (IG4)<br>T_g: 225°C	Re-Ir coating<br>Thickness: 250 nm	Temperature: 320°C<br>Pressure: 1.18 MPa	Isothermal molding	9 nm	IR imaging applications
Wang et	Precision	Aspherical lens	B270	N/A	Temperature: 635°C	Isothermal	2 nm	Light

al./Ohio State University [87]	grinding		T_g: 533°C		Pressure: 1.58 MPa	molding		manipulation for optical systems
Bernhardt et al./Fraunhofer Institute for Production Technology, Germany [88]	Grinding and polishing	Lens array	P-LASF47<br>T_g: 493 °C	ta-C DLC coated<br>Thickness: 300 nm	Temperature: 590°C<br>Pressure: 3 kPa	Isothermal molding	5 nm	Optical applications
Allen et al./Ohio State University [89]	Grinding and polishing	Aspherical lens	BK7<br>T_g: 557 °C	Pt coating<br>Thickness: 500 nm	Temperature: 684°C<br>Pressure: 4 MPa	Isothermal molding	2 nm	Optical applications
Allen et al./Ohio State University [90]	Grinding and polishing	Aspherical lens	BK7<br>T_g: 557°C	Pt coating<br>Thickness: 500 nm	Temperature: 700°C<br>Pressure: 4 MPa	Isothermal molding	2 nm	Optical applications
K. Jiang et al./College of Mechatronic	Wire-cur EDM	Microfluidic channel	BK7<br>T_g: 560°C	Nitride coating	Temperature: 660°C<br>Pressure: 4 MPa	Hot embossing	Surface roughness (S_a): 1.1 nm	Microfluidic application

s and Control Engineering/ China [91]								
K. Liet al./University of Science and Technology Liaoning/ China [92]	Micro-EDM	Microholes Diameter: 320 μm Period: 320 μm	D-K9 T_g: 496°C	Nitride coating Thickness: 12 nm	Temperature: 540°C Pressure: 12 MPa	Hot embossing	Surface roughness: N/A	Optical application

Table S5: Summary of studies of GC micro/nano mold insert systems for glass imprinting. Acronyms: MLA: microlens array; RMS: root mean square; MEMS: microelectromechanical system; ICP: inductively coupled plasma; RIE: reactive ion etching; FIB: focused ion beam; N/A: not available.

Authors & Affiliations	Mold fabrication methods	Pattern geometries on mold	Glass materials	Anti-adhesion coating materials	Molding conditions	Molding methods	Mold surface roughness	Proposed/ tested applications
Tseng et al./National Changhua University of Education, Taiwan [23]	Laser micromachining	Microchannel<br>Width: 120 μm<br>Depth: 180.26 μm<br>Length: 10 mm	Soda-lime glass<br>T_g: 564°C	N/A	Temperature: 660°C<br>Pressure: 0.22 kPa	Isothermal molding	Surface roughness (R_a): 672 ± 80 nm	Microfluidic applications
Youn et al./National Institute of AIST, Japan [105]	Laser machining and FIB milling	Various microstructures	Pyrex glass<br>T_g: 560°C<br><br>Quartz glass<br>T_g: 1200°C	N/A	Temperature: 645°C<br>Pressure: 2.83 kPa<br><br>Temperature: 1305°C<br>Pressure: 0.22 kPa	Isothermal molding	Surface roughness (R_a): 80 nm	Optical applications
Sasaki et al./Advanced Manufacturing Research Institute	Dicing/sawing<br><br>Laser machining	Square blocks<br>Size: 100 μm× 100 μm× 50 μm<br><br>Microchannel	Borofloat glass<br>T_g: 525°C	N/A	Temperature: 655°C<br>Pressure: 2 MPa<br><br>Temperature: 655°C<br>Pressure: 13 MPa	Isothermal molding	Surface roughness (R_a): 300 nm	Bio-MEMS applications

(AIST), Japan [106]								
Takahashi et al./National Institute of AIST, Japan [107]	Dicing/sawing	Various microstructures Height and width 100 μm, 100 μm, 50 μm, 50 μm, 50 μm, 100 μm respectively	Pyrex glass T_g: 560°C	NA	Temperature: 645°C Pressure: 2.83 MPa	Isothermal molding	Surface roughness (R_a): 150 nm	Optical applications
Ito et al./Shinshu University, Japan [108]	Dicing/sawing	Line and space pattern Width: 10 μm, Period: 20 μm Height: 11.5 μm	Pyrex T_g: 560°C<br><br>D263 T_g:557°C	N/A	Temperature: 670°C Pressure: 3.56 MPa<br><br>Temperature: 620°C Pressure: 3.56 MPa	Isothermal molding	Surface roughness: optical quality	Optical applications
Takahashi et al./University of Shinshu [109]	FIB milling	Various microstructures 10×10×2μm³ 10×10×3 μm³ 10×10×7μm³ Micro pyramid: Width: 20 μm Height: 15 μm Line-and-space pattern: Depth: from 1 μm to 300 nm	Pyrex glass T_g: 460°C<br><br>Quartz glass T_g: 1200°C	NA	Temperature: 590°C, 595°C, and 590°C Pressure: 0.22 MPa, 0.45 MPa, and 0.22 MPa<br><br>Temperature: 1385°C Pressure: 0.43 MPa	Isothermal molding	Surface roughness (R_a): 20 nm	MEMS applications

Takagi et al./Advanced Manufacturing Research Institute, National Institute of AIST, Japan [110]	FIB milling	Line-and-space pattern<br>Width: 5 µm, 1 µm, 0.5 µm, and 0.3 µm<br>Height: 150 nm	B207<br>T_g: 521°C	N/A	Temperature: 440°C<br>Pressure: 0.01 MPa<br><br>Temperature: 550°C<br>Pressure: 1 MPa	Active heating<br><br>Isothermal molding	NA	MEMS applications
Youn et al. /National Institute of AIST, Japan [111]	FIB milling	Square pattern<br>20 µm × 20 µm<br><br>Fluidic channel	Pyrex Glass<br>T_g: 560°C	N/A	Temperature: 645°C<br>Pressure: 2.83 MPa<br><br>Temperature: 1300°C<br>Pressure: 0.26 MPa	Isothermal molding	Surface roughness (R_a): 5 nm	MEMS applications
Chen et al./Ohio State University, USA [112]	Photolithography and RIE	Microholes<br>Diameter: 100 µm<br>Depth: 10 µm<br>Pitch: 200 µm	P-SK57<br>T_g: 493°C	N/A	Temperature: 565°C<br>Pressure: 1 MPa	Isothermal molding	NA	Optical intensity modulation
Yasui et al./Kanagawa Industrial Technology Center,	EBL and RIE	Line and space patterns<br>Width: 500 nm<br>Height: 300 nm	Pyrex glass<br>T_g: 560°C	NA	Temperature: 630°C<br>Pressure: 0.31 MPa	Isothermal Molding	Surface roughness (R_a): 7.792 nm	Optical applications

Japan [113]								
Mekaru et al./National Institute of AIST, Japan [114]	Photolithography and RIE	Circular/square pattern:<br>Diameter/width: 5 μm<br>Depth: 2.1 μm<br>Vertical sidewall: 2.38<br><br>Circular/square pattern:<br>Diameter/width: 5 μm<br>Depth: 0.8 μm<br>Curved sidewall: 6.25	Pyrex glass<br>T_g: 560°C<br><br>Quartz glass<br>T_g: 1150°C	NA	Temperature: 645°C<br>Pressure: 3.11 MPa<br><br>Temperature: 1320°C<br>Pressure: 2 MPa	Isothermal molding	Surface roughness (R_a): 1–3 nm	MEMS applications
Ju et al./Chung-Ang University, Korea [116]	Carbonization of patterned polymer replicated from a Si master mold	Micrograting pattern<br>Pitch: 46.3 μm,<br>Height: 3.81 μm	K-PG375<br>T_g: 344°C	N/A	Temperature: 380°C<br>Pressure: 3 MPa	Isothermal molding	RMS roughness (R_z): 3.7 nm	Optical applications
Kim et al./Chung-Ang University, Korea [117]	Carbonization of patterned polymer replicated from a Ni master mold	Fresnel lens<br>40×40 mm^2	Soda-lime glass<br>T_g: 564°C	NA	Temperature: 720°C<br>Pressure: 140 kPa	Isothermal molding	Average roughness (R_a): 16 ± 2 nm	CPV

Ito et al./Shinshu University, Japan [119]	FIB milling	Line and space pattern<br>Width: 723 nm<br>Height: 734 nm<br><br>MLA<br>Sag height: 0.84 μm	D263<br>T_g: 557°C	N/A	Temperature: 670°C<br>Pressure: 7.3 MPa<br><br>Temperature: 620°C<br>Pressure: 4 MPa	Isothermal molding	NA	Optical applications
Mori et al. /National Institute of AIST, Japan [120]	EBL and plasma-based RIE	Pattern structure 1:<br>Period500 nm and height 730 nm<br>Width: 150, 220, 290, and 330 nm<br>Pattern structure 2:<br>Pitch 500 nm and depth 350	U-SK55M<br>T_g: 430°C	N/A	Temperature: 500°C<br>Pressure: 17.78 kPa	Isothermal molding	NA	Optical polarization
Prater et al./École Polytechnique Fédérale de Lausanne (EPFL), Switzerland[	Photolithography and ICP RIE	DOE<br>Height: 690 nm<br>Critical dimension: 2 μm	L-BAL42<br>T_g: 506°C	N/A	Temperature: 550°C<br>Pressure: 80 kPa	Isothermal molding	Surface roughness (R_a): 2.5 nm	Beam splitting

121]								
Chenet al./Ohio State University, USA [122]	Micromachining and dry etching	DOE<br>Height: 150 nm<br>Width: 3 μm	B207<br>T_g: 533°C	N/A	Temperature: 690°C<br>Pressure: 400 kPa	Isothermal molding	Surface roughness (R_a): 40 nm	Laser light intensity modulation
Prateret al./EPFL, Switzerland [123]	Photolithography and RIE	DOE pattern<br><br>Square pattern: 20μm×20μm<br>Height: 600 nm	L-BAL42<br>T_g: 506°C<br><br>Fused Silica<br>T_g: 1200°C	N/A	Temperature: 550°C<br>Pressure: 4.15 MPa<br><br>Temperature: 1360°C<br>Pressure: 4.15 MPa	Isothermal molding	Surface roughness (R_a): 3 nm	DOE
Ikeda et al./Hokkaido University, Japan [124]		Square pit 10mm×10mm×1mm	K-PSK200<br>T_g: 387°C<br><br>L-BAL42<br>T_g: 504°C	N/A	Temperature: 420°C<br>Pressure: 2 MPa<br><br>Temperature: 560°C<br>Pressure: 2 MPa	Isothermal molding	Surface roughness (R_a): 1.3 nm	Optical imaging
Youn et al./National Institute of AIST, Japan [125]	EBL and RIE	Microfluidic channel<br><br>Line and space pattern<br>Period: 1 μm<br>Depth: 900 nm	Quartz glass<br>T_g: 1200°C<br><br>Borosilicate glass<br>T_g: 557°C	N/A	Temperature: 1300°C<br>Pressure: 3.05 MPa<br><br>Temperature: 650°C<br>Pressure: 1.33 MPa	Isothermal molding	Surface roughness (R_a): 2 nm	Optical and microfluidic applications
Haq et	Carbonization of	Microchannel	Soda-lime	NA	Temperature: 720°C	Isothermal	12 ± 1 nm	Microfluidic

al./Chung-Ang University, Korea [126]	patterned polymer replicated from a Si master mold	Pitch: 396.7 µm<br>Height: 33.3 µm	glass<br>T_g: 564°C		Pressure: 10 kPa	molding		applications
Kim et al./Chung-Ang University, Korea[127]	Carbonization of patterned polymer replicated from a Si master mold	MLA<br>Pitch: 9.9 µm,<br>Sag height: 0.704 µm	Soda-lime glass<br>T_g: 564°C	NA	Temperature: 720°C<br>Pressure: 2 kPa	Isothermal molding	RMS roughness (R_z): 4.78 nm	Optical intensity modulation
Janget al./Chung-Ang University, Korea [128]	Carbonization of patterned polymer replicated from a Si master mold	Microfluidic channel<br>Orifice: 43.5 µm<br>Width: 88.93 µm<br>Height: 28.07 µm	Soda-lime glass<br>T_g: 564°C	NA	Temperature: 680°C<br>Pressure: 163.2 kPa	Isothermal molding	Average roughness (R_a): 2.83 ± 0.41 nm	Microfluidic applications
Janget al./Chung-Ang University, Korea [129]	Carbonization of patterned polymer replicated from a Si master mold	Microchannel<br>Width and height of inlet, outlet and extraction channels are 153.6 µm, 51.6 µm, 462.7 µm, 53.7 µm, 151 µm 53.4 µm, respectively	Soda-lime glass<br>T_g: 564°C	NA	Temperature: 700°C<br>Pressure: 18 kPa	Isothermal molding	RMS roughness (R_z): 12 nm	Microfluidic applications
Haq et al./ Chung-Ang	Carbonization of patterned	Nanopin array<br>Period: 325 µm,	Soda-lime Glass	N/A	Temperature: 700°C<br>Pressure: 1 MPa	Isothermal molding	N/A	Antireflection

University, Korea [130]	polymer replicated from a Si master mold	Diameter: 110 μm Depth: 220 μm	T_g: 564°C					

Table S6: Summary of studies on other micro/nano mold insert systems for glass imprinting. Acronyms: MLA: microlens array; KrF: krypton fluoride; RIE: reactive ion etching; FIB: focused ion beam; CBG: carbon-bonded graphene; DLC: diamond-like carbon; Si_3N_4: silicon nitride; DOE: diffractive optical element, AAO: Anodic aluminum oxide.

Authors & Affiliations	Mold fabrication methods	Pattern geometries on the glass	Glass materials	Anti-adhesion coating materials	Molding conditions	Molding methods	Mold surface roughness (R_a)	Proposed/tested applications
Zhu et al./Zhejiang University of Science and Technology, China[132]	CC<br><br>Ultraprecision diamond grinding	Aspherical lens Concave radius: 97.01 mm Center thickness: 7 mm	Chalcogenide glass (NBU-IR1) T_g: 285°C	DLC coating	Temperature: 315°C Pressure: 0.7 MPa	Isothermal molding	80 nm	IR imaging
Zhang et al./Ohio State University, USA [134]	Al alloy mold<br><br>Servo-assisted ultraprecision diamond machining	Sag height: 5.22 µm	Chalcogenide glass (As_2Se_3) T_g: 197°C	NA	Temperature: 215°C Pressure: 2.7 MPa	Isothermal molding	13.65 nm	IR imaging applications

L. Zhang et al./Ohio State University, USA[135]	Al alloy mold<br><br>Multi-axes ultraprecision diamond machining	Freeform MLA Dimension: 2.5×2.5 mm^2	Chalcogenide glass (As_2Se_3)	NA	Temperature: 225°C<br>Pressure: 8.86 kPa	Isothermal molding	22.64 nm	Infrared imaging
Komori et al./Kyoto University, Japan [136]	CVD diamond mold<br><br>FIB milling	Various microstructures<br><br>Line and space patterns: 8μm×50μm Height: 0.17μm to 3.60μm. 5μm×50μm, 3μm×30μm 1μm×10μm Height: 2μm and 1μm Square pits 8μm×10μm and1μm×10μm Depth: 0.36μm and0.31μm	Pyrex glass T_g: 560°C<br><br>BK7 glass T_g: 560°C<br><br>Tempaxglass T_g: 525°C	No coating	Temperature: 690°C<br>Pressure: 1 MPa<br><br>Temperature: 650°C<br>Pressure: 1 MPa<br><br>Temperature: 640°C<br>Pressure: 1 MPa	Isothermal molding	Very low	Biomedical applications
Hirai et	Si-based SiO_2	Nanograting	Low Tg	Si_3N_4	Temperature: 405°C	Isothermal	0.251–0.288	Optical

al./Osaka Prefecture University, Japan [138]	mold<br><br>KrF lithography and RIE	pattern<br>Width: 1.0 μm, 330 nm, and 250 nm<br>Depth: 300 nm	glass<br>T_g: 375°C	coating<br>Thickness: 20 nm	Pressure: 45 MPa	molding	nm	applications
Li et al./Ohio State University, USA [139]	Fused silica mold<br><br>Photolithography and RIE	Square pit<br>Size: 10 μm × 10 μm<br>Height: 0.8 μm	Arsenic trisulfide glass ($As_{40}S_{60}$)<br>T_g: 180°C	CBG coating	Temperature: 271°C<br>Pressure: 25 kPa<br><br>Temperature: 240°C<br>Pressure: 33.6 kPa	Active and isothermal molding	3 nm	Camera applications
Arai et al./Shinshu University, Japan [140]	CC	Spherical lens<br>Diameter: 7.2 mm	BK7<br>T_g: 572°C	NA	Temperature: 670°C<br>Pressure: 20 MPa	Isothermal molding	20 nm	Optical imaging
X. Liu et al./ Shenzhen University, China[141]	AAO	Nano porous patterns<br>Diameter: 100 nm to 250 nm	D-FK61<br>T_g: 440°C	NA	Temperature: 470°C<br>Pressure: 12.7 MPa	Isothermal molding	NA	Antireflection
Feng Y. et al/ Shenzhen University, China[142]	AAO	Nano porous patterns<br>Diameter: 390 nm	D-FK61<br>T_g: 440°C	NA	Temperature: 480°C<br>Pressure: 12.7 MPa	Isothermal molding	NA	Antireflection

Yiet al./Ohio State University, USA[143]	Fused silica mold Photolithography and RIE etching	Lateral: 10 μm Height: 330 nm	K-PG325 T_g: 285°C	NA	Temperature: 325°C Pressure: 0.13 MPa	Isothermal molding	24 nm	DOE applications
